# Reducing COVID-19 Vaccine Hesitancy by Implementing Organizational Intervention in a Primary Care Setting in Bahrain

**DOI:** 10.7759/cureus.19282

**Published:** 2021-11-05

**Authors:** Rabih Abou Leila, Mustafa Salamah, Sarah El-Nigoumi

**Affiliations:** 1 Family Medicine, American Mission Hospital, Manama, BHR; 2 Pediatrics, American Mission Hospital, Manama, BHR

**Keywords:** patient education, patient counseling, vaccine hesitancy, herd immunity, covid-19 vaccine

## Abstract

Background: Refusal to receive the COVID-19 vaccine or hesitancy is a global threat. Hence, reducing vaccine hesitancy is the next challenge for policymakers and the healthcare system. However, people trust healthcare professionals more than any other source of information. Accordingly, planning for an effective process and competent healthcare professional to elicit and address the patient's concerns is an imperative measure. This quality improvement project aimed to improve physicians’ COVID-19 vaccine advice to reduce vaccine hesitancy.

Methods: The study used judgmental sampling and involved 665 hesitant patients over 19 weeks. The team utilized the Plan-Do-Study-Act method to implement COVID-19 vaccine physicians’ reminders and upgrades physicians’ communication skills to conduct an effective tailored communication that addresses the patient’s concerns toward COVID-19 vaccines. The team used pre-post design to evaluate the impact of counseling on the hesitancy rate of patients before and after the intervention over time. The main outcomes were the percentage of physicians’ COVID-19 vaccine advice for patients and the percentage of hesitancy rate before and after implementation.

Results: There were 665 hesitant patients before intervention. However, after the intervention, the number decreased to 193 patients, and the control chart revealed a reliable process. The percentage of recommendations by physicians has increased from 1% to 51% after 19 weeks of implementation and with a controlled process.

Conclusion: The study has found that rectifying process barriers and upgrading physicians’ skills would improve the COVID-19 vaccine counseling rate and introducing tailored communication would reduce the hesitancy rate. Nevertheless, the study was constrained by a lack of information on the impact of social media and national measures on patients’ decisions. Additional studies with more emphasis on other patients’ behavior determinants are necessary.

## Introduction

Herd immunity is critical to control the COVID-19 pandemic [1]. It can be achieved when a large number of individuals in societies have immunity against the COVID-19 [1,2]. However, vaccine hesitancy is a substantial threat to vaccination efforts. In the United Kingdom, 31% of surveyed people have vaccine hesitancy [3,4]. On January 31, 2021, despite the availability of vaccines, only 10% of the population received the first dose of the COVID-19 vaccine in the Kingdom of Bahrain [5]. Therefore, the entire population was not fully immunized. Moreover, a local survey conducted between November and December 2020 in the authors’ hospital revealed that 46% of patients have concerns about the COVID-19 vaccine. Hence, addressing the concerns is a prerequisite to reducing the vaccination hesitancy and increasing the immunization rate [6,7]. However, rectifying the vaccine hesitancy mandates interventions at the national, community, and healthcare organization levels [8]. Although national and community interventions are useful, healthcare measures are the most effective action in the mass vaccination endeavor [8]. Physicians play a key role in counseling the patients about COVID-19 vaccines options, risks and benefits, and side effects. However, according to a peer review report in the hospital, <1% of patient charts documented COVID-19 counseling. This quality improvement project aimed to improve the COVID-19 vaccine counseling and evaluate its effects on vaccine hesitancy among patients by comparing the rates before and after the intervention. The hypothesis was that the counseling would bring about a positive outcome. Accordingly, an implementation team has been created to lead this project including two family physicians and four nurses. Throughout this paper, the term team is used to refer to the implementation team.

## Materials and methods

This study was conducted between January 2021 and May 2021 at primary care hospital with eleven doctors and fourteen nurses. The center was purposively selected because manages approximately 1800 patients per month who are entitled to receive the COVID-19 vaccines. The study was approved by the ethical committee in the hospital (Date: December 26, 2020, ID 04/2021).

According to Deming, understanding the system is an obligatory step prior to embarking on the improvement initiative [9]. According to the team, the current system holds flaws at three distinct levels: organizational defects (no system reminders at points of care, no adoption of policy); interpersonal defect (no communication training strategies); and individual defect (the physician is not aware of latest guidelines, the patient has multiple questions about the vaccine). The evidence-based strategies suggest a multilevel intervention to face the COVID-19 hesitancy, which includes the following: (1) organizational intervention; (2) interpersonal intervention; and (3) individual intervention [7,10].

Regarding implementation of the interventions, the team established an implementation plan with the help of the model for improvement (MFI) [11]. The evidence from previous studies showed that the MFI is an effective implementation method in the healthcare industry [12]. The team considered a large-scale implementation in the clinic because the staff believed in the project and failure cost is low [13]. Accordingly, three Plan-Do-Study-Act (PDSA) cycles were conducted to attain the aim: PDSA one: addressing process barriers; PDSA two: upgrading the physicians’ knowledge and communication skills; and PDSA three: patient education. Various PDSA cycles were simultaneously run at the commencement of January 2021.

Participants were involved in the implementation stage of this project. The team used judgmental sampling [13] to recruit the patients. Enlightened with the Shewhart sampling principle, “We need just enough observational data to tell us if our systems of production are stable or unstable” [14]. The subject matter expert decided to include only five patients per day in 19 weeks. The inclusion criteria include the participant should be aged > 18 years and eligible for the vaccine, did not take any vaccine dose, and has COVID-19 vaccine hesitancy. The hesitant patients were identified by asking the eligible patients during nursing triage. The nurse must ask the potential participant about COVID-19 vaccine the following question: “COVID-19 vaccines are available; are you planning to get the vaccine?” If the patient's reply was one of the following: may take it; uncertain; possibly not to take the vaccine, or absolutely not to avail the vaccine, then the patient is considered hesitant to receive the vaccine [15]. Then the nurse notifies the patient about the project and outcome measure and takes verbal consent to participate in the study. A total of 665 individuals were selected and met the criteria.

The medical charts of the selected participants were flagged by nurses indicating that patients need counseling for COVID-19 vaccine. Then the physician receives the hesitant patient’s file and prepares the consultation agenda that includes counseling about the vaccine. The physicians were informed ahead about COVID-19 vaccines dilemmas through a lecture [16] and trained to use the Ask-Offer-Ask strategy [7] to conduct a tailored communication about COVID-19 concerns with hesitant patient [17]. During the consultation, the physicians ask the patients “What do you already know about the COVID-19 vaccine?” and “What ideas do you have?” Then, the physicians offer a strong recommendation and tailored communication. Then, the physicians ask the patient again, “How does the recommendation resonate with you?” This strategy is selected because of its effectiveness [8]. The physicians must document the counseling in the patient’s record by selecting suitable International Statistical Classification of Diseases and Related Health Problems (ICD 10) Z71.8, which stands for specified counseling for COVID-19 [18]. After completion of the physician’s counseling, one investigator interviewed the selected patients, seeking information about the physician counseling and whether they are planning to receive the vaccine after counseling. The physicians were receiving performance feedback regularly from the team.

The study assessed the impact of counseling on the hesitancy rate of patients before and after the intervention over time. We selected two measures including (1) percentage of patients who have COVID-19 vaccine hesitancy (outcome measure) and (2) percentage of compliance of physicians with COVID-19 vaccine counseling (process measure). The findings were presented using statistical process control charts [14]. We utilized Nelson’s guidelines to differentiate between the variations, either special or common [19] since the improvement scale is related to the variant type. While assignable cause necessitates inquiry of the unpredictable cause and comprised the adjustment, the common variation suggests stability of the process [19]. The information was presented in a Microsoft Excel table in compliance with the confidentiality policy, and data analysis was conducted by the study group with the assistance of QI Macros software (2017).

## Results

Physicians’ compliance with counseling

**Figure 1:**
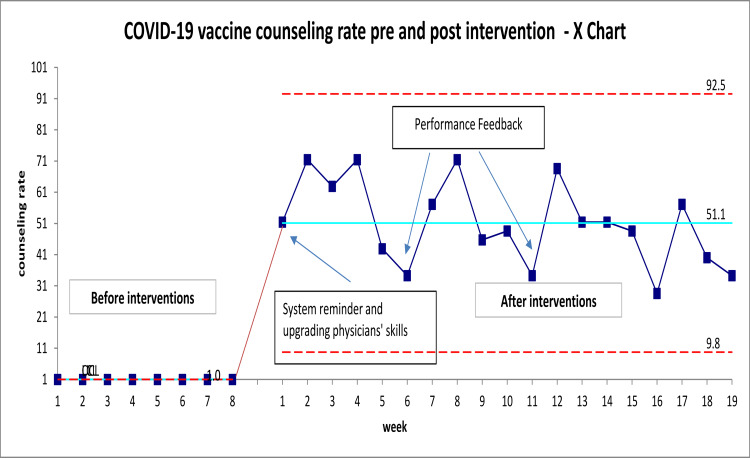
Control chart COVID-19 vaccine counseling rate pre- and post-intervention

The goal was to improve physicians’ compliance with COVID-19 vaccine counseling from 1% to 50% at the end of May 2021. Following the implementation of several changes (implementing the flagging system, CME, communication strategies training, and feedback evaluation), the primary care physician provided counseling to 340 of 665 patients in the intervention group. Hence, the documented COVID-19 counseling for hesitant patients has increased from 1% (baseline) to 51% after 19 weeks of implementation. The control chart in Figure 1 highlights the performance of physicians over time following the introduction of changes. As shown in the graph, the documented compliance of physicians with counseling in November and December 2020 was only 1%. However, on week one, following the introduction of changes, a phenomenal increase in compliance was noted and reached 51%, exceeding the target. Moreover, the graph shows the common cause variation, indicating stability of the process. Hence, the latest changes helped to create an efficient process, enabling patient counseling.

Hesitancy rate results

**Figure 2:**
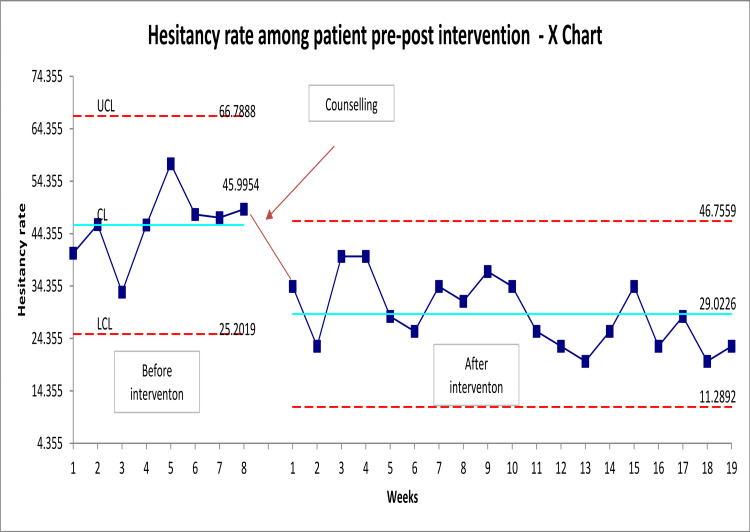
Control chart COVID-19 vaccine hesitancy rate pre- and post-intervention

The goal was to reduce the COVID-19 hesitancy rate among patients who have concerns about the vaccine from baseline data 46% to 30% after the intervention. Following the implementation of counseling and patient education, the hesitant patient’s number has decreased from 665 to 193. The control chart in Figure 2 highlights the hesitancy rate over time following the introduction of changes. The graph demonstrates a comparison between pre- and post-intervention. According to the graph, the hesitancy rate 8 weeks before the intervention was 46%, while, after the intervention, the average was 29% in the post-intervention. Moreover, the graph shows the common cause variation, indicating the stability of the process. Hence, we can conclude that physicians’ counseling and provision of patient education helped a good number of patients to change their mind toward COVID-19 vaccine.

## Discussion

COVID-19 vaccine hesitancy is a complex problem requiring coordinated effort to improve the uptake rate [7]. In this study, the team approached the hesitancy problem at the primary healthcare level by instituting multifaceted changes, including organization, interpersonal and individual target of the system, communication skills, and knowledge of the physician and patient. These interventions were launched simultaneously. This study aimed to assess the impact of various interventions on patients’ hesitancy.

The absence of reminder alerts, lack of process that recognizes patients with COVID-19 vaccine hesitancy, and inadequate feedback were the contextual system barriers that affect the counseling rate by physicians to patients and consequently reduce the uptake rate of COVID-19 vaccines. Conjointly, the literature provided evidence for effectiveness of prompt point of care in improving the vaccine counseling rate by physicians [20]. Moreover, the available evidence proves the positive impact of physicians’ knowledge [21] and performance feedback on the compliance of physicians with guidelines [22]. The current study found that several changes in process changes and continuous learning improve the compliance rate. Therefore, it can be assumed that several changes that address organizational barriers help improve the compliance of physicians with the guidelines.

Furthermore, studies showed that patients who obtained information from the physician had less vaccine hesitancy [23]. However, many researchers report that addressing vaccine hesitancy requires more tailored communication than providing numbers and facts [24]. Moreover, according to the literature, patient education materials improve the patient’s attitude toward vaccination [25]. In the project site, the vaccine hesitancy rate reached 46% before the intervention. While the average of hesitant patients decreased to 29% post-intervention. The results in this project showed the importance of tailored communication and patient education material in improving the acceptance of vaccines. These results matched the earlier studies [25,26]. Therefore, it can be assumed that addressing patient concerns about the medical condition through tailored communication would improve healthcare decisions among patients.

Limitation of the study

Although the findings reported here shed new light on the impact of counseling on patients’ decisions, the study did not include the impact of the Internet and national measures on patients’ behavior after consultation. Moreover, the study proved the importance of organizational changes to improve the counseling rate by the physician. However, the mentioned changes were contextual factors.

## Conclusions

Reducing COVID-19 vaccine hesitancy is critical in controlling the pandemic. In this project, we addressed the healthcare organizational barriers that would improve the hesitancy status. Rectifying the system problems and upgrading the physicians’ skills and knowledge elicited the vaccine concerns from the hesitant patient and helped in conducting tailored counseling. The current study illustrated that appropriate counseling was associated with lower hesitancy rates. This study lays the groundwork for future research about making better health decisions through tailored communication. However, the study is limited by the lack of a control group for examining the confounding factors. A further study with more focus on the impact of national measures and social media on patients’ behavior is required.
